# Use of Ceftaroline Fosamil in Osteomyelitis: CAPTURE Study Experience

**DOI:** 10.1186/s12879-019-3791-z

**Published:** 2019-02-21

**Authors:** Leonard B. Johnson, Ananthakrishnan Ramani, David J. Guervil

**Affiliations:** 1 Ascension St. John Hospital and Medical Center and Wayne State University School of Medicine, 19251 Mack Avenue, Suite 340, Grosse Pointe Woods, MI 48236 USA; 2 Columbia Memorial Hospital, Hudson, NY USA; 3 Memorial Hermann-Texas Medical Center, Houston, TX USA

**Keywords:** Gram-positive osteomyelitis, Ceftaroline fosamil, Parenteral antibiotics, Off-label therapy

## Abstract

**Background:**

Osteomyelitis is often challenging to treat. This analysis examined the clinical experience of patients with gram-positive osteomyelitis treated with ceftaroline fosamil in the phase 4 Clinical Assessment Program and Teflaro® Utilization Registry (CAPTURE) study.

**Methods:**

Data including patient demographics, past illnesses, risk factors, disease characteristics, antibiotic use, pathogens isolated, and clinical outcome were collected between September 2013 and February 2015 by review of randomly ordered patient charts from participating sites in the United States. Clinical success was defined as discontinuation of ceftaroline fosamil following clinical cure with no further need for antibiotics or clinical improvement with switch to another antibiotic treatment.

**Results:**

A total of 150 patients with gram-positive osteomyelitis were treated with ceftaroline fosamil. Most patients (117/150; 78.0%) were treated with 600 mg ceftaroline fosamil per dose; 143/150 patients (95.3%) received a dose every 12 h. The majority (89/150 patients; 59.3%) had been previously diagnosed with diabetes mellitus or peripheral arterial disease. Osteomyelitis was associated with hardware in 32/150 patients (21.3%). Methicillin-resistant and methicillin-susceptible *Staphylococcus aureus* (MRSA; MSSA) were the most commonly isolated pathogens, observed in 93/150 (62.0%) and 21/150 (14.0%) patients, respectively. Clinical success with ceftaroline fosamil therapy was observed in 139/150 (92.7%) patients overall, 81/89 (91.0%) patients with diabetes or peripheral arterial disease, and 18/20 (90.0%) patients who had hardware implanted before ceftaroline fosamil therapy (none had hardware removed during therapy). Patients who received prior antibiotic therapy or ceftaroline fosamil as monotherapy experienced clinical success rates of 93.9% (107/114) and 91% (91/100), respectively. Among patients who received concurrent antibiotic therapy, the clinical success rate was 96.0% (48/50). Patients who were infected with MRSA or MSSA had clinical success rates of 92.5% (86/93) and 100% (21/21), respectively. A total of 2/150 (1.3%) patients discontinued ceftaroline fosamil therapy because of adverse events.

**Conclusions:**

Clinical success rates with ceftaroline fosamil were high in patients with gram-positive osteomyelitis, including those with diabetes or peripheral arterial disease and those with MRSA or MSSA.

**Electronic supplementary material:**

The online version of this article (10.1186/s12879-019-3791-z) contains supplementary material, which is available to authorized users.

## Background

The annual incidence of osteomyelitis is estimated to be approximately 22 cases per 100,000 person-years, with the rate increasing with age [1]. Acute osteomyelitis is characterized by bone alterations caused by pathogenic bacteria occurring within approximately 2 weeks of infection onset, whereas chronic osteomyelitis may be characterized by bone necrosis and occurs approximately 6 weeks after infection onset [2, 3].

In most adult cases, osteomyelitis results from a direct inoculation of bacteria into the bone (exogenous osteomyelitis) due to trauma or surgery, from an adjacent source of infection such as an infected ulcer, or from hematogenous seeding [3]. Inflammation contributes to the tissue necrosis and bone destruction by causing compression of the vascular network and subsequent ischemia [4]. Development of an avascular area prevents antibiotics and inflammatory cells from reaching the site of infection, complicating medical treatment and contributing to the relatively high rate of treatment failure compared with other types of infection [4, 5].

*Staphylococcus aureus* is the most common pathogen isolated from bone and prosthetic joint infections, found in approximately 40% of cases, with methicillin-resistant *S aureus* (MRSA) becoming increasingly more common [1–3]. Approximately 20% of patients with diabetic foot infection have underlying osteomyelitis and are therefore at risk for lower extremity amputation [6, 7]. Peripheral neuropathy and peripheral arterial disease (PAD) contribute to the increased risk of osteomyelitis in patients with diabetes mellitus (DM) by causing infections to go unnoticed and impeding the healing process, respectively [2].

Unlike infections of prosthetic joints and chronic osteomyelitis, which often require combined medical and surgical therapy, use of antimicrobial therapy alone is generally adequate for the treatment of acute osteomyelitis [4]. The standard recommendation for the treatment of chronic osteomyelitis is 4 to 6 weeks of parenteral antibiotics, with a cure rate of approximately 60–90% in cases treated with parenteral β-lactam antibiotics; however oral therapy with agents that have high bioavailability can be used as an alternative to parenteral therapy [8]. Comorbid conditions such as DM and PAD contribute to a higher risk of recurrence [9]. Bone penetration of β-lactam antibiotics ranges from 5 to 20% of serum levels, but active levels of antibiotics are typically sufficient to exceed pathogen minimum inhibitory concentration (MIC) when delivered parenterally [8].

Ceftaroline fosamil is a cephalosporin that has activity against gram-positive and gram-negative bacteria; ceftaroline inhibits bacterial cell wall synthesis by binding to penicillin-binding proteins (PBPs) [10]. The high affinity of ceftaroline for PBP2A and PBP2x helps explain its activity against MRSA and multiple-drug resistant *Streptococcus pneumoniae*, respectively [11]. Ceftaroline has been shown to rapidly penetrate into the cancellous and corticol bone of patients undergoing hip replacement surgery [12]. Ceftaroline fosamil has been approved for treatment of acute bacterial skin and skin structure infections (ABSSSIs) [10] based on the success of the phase 3 clinical trials, CANVAS 1 and CANVAS 2 [13, 14], and is the first cephalosporin to be used in the United States with activity against MRSA (MIC for 90% of isolates = 1 μg/mL) [15].

Several recent case reports have described success in the treatment of osteomyelitis with ceftaroline fosamil [16, 17], as have large retrospective observational studies [18, 19]. This retrospective registry study sought to evaluate the experience of patients with osteomyelitis treated with ceftaroline fosamil in Clinical Assessment Program and Teflaro® Utilization REgistry (CAPTURE), a phase 4, multicenter, retrospective cohort study designed to describe the contemporary clinical use of ceftaroline fosamil, including off-label use, in the United States [20].

## Methods

### Study design and patients

For the present analysis, CAPTURE data were collected from participating centers by review of randomly ordered patient charts between September 2013 and February 2015 as part of the extended CAPTURE study [20]. Male and female patients ≥18 years of age with a primary diagnosis of gram-positive osteomyelitis who received four or more consecutive intravenous doses of ceftaroline fosamil, including a final dose ≥30 days before the start of data collection, were eligible for inclusion in the analysis. Gram-positive osteomyelitis was defined as an infection of bone with imaging compatible with such a diagnosis and a corresponding culture for a gram-positive pathogen obtained intraoperatively or for coagulase-negative staphylococci obtained intraoperatively and associated with hardware. Gram-positive osteomyelitis secondary to gram-positive bacteremia was not included. The following exclusion criteria were used for this analysis: missing hospital admission/discharge or ceftaroline fosamil treatment information, previous chart data extraction for CAPTURE, diagnosis with more than one of the specified infectious diseases within 96 h before the start of ceftaroline fosamil or during treatment, or infection with a pathogen for which ceftaroline fosamil monotherapy is inappropriate.

### Data collection and analysis

Data collected for each enrolled patient included demographics and baseline characteristics, site of care, relevant medical and surgical history (including past illnesses), disease characteristics (including risk factors, complications, site of infection, laboratory results, and previous antibiotic treatment), pathogen characteristics, ceftaroline fosamil usage information (including location of care), and clinical outcome. Clinical outcome was defined as either clinical success or clinical failure and determined based on individual physician assessment. Clinical success was defined as clinical cure with no further need for antibiotic or clinical improvement with switch to another antibiotic, after discontinuation of ceftaroline fosamil. For clinical failure, the reason for discontinuation of ceftaroline fosamil was an adverse event (AE) or switch to another IV antibiotic because of insufficient therapeutic effect. If there was not enough information to determine the outcome, the outcome was classified as indeterminate and the patient was not included in the evaluation. Patient records were reviewed through the completion of antibiotic therapy discontinuation for outcome and safety assessments by each center’s investigative site personnel, based on pharmacy listings. This study was submitted to and approved by appropriate institutional review boards (IRBs) as listed in Additional file 1: Table S1. This study was conducted in compliance with the International Conference on Harmonisation (ICH) Technical Requirements for Registration of Pharmaceuticals for Human Use: Guidance for Industry E6 Good Clinical Practice: Consolidated Guidance (1996), Good Pharmacoepidemiology Practice, Health Insurance Portability and Accountability Act, 21 CFR Part 11, and any additional national or IRB requirements. Names of primary investigators and IRBs are listed in Additional file 1: Table S1. Chart abstraction was performed ≥30 days after ceftaroline fosamil administration to ensure retrospective collection of data. Due to the retrospective nature of data collection for this study, patient written consent was waived or collected before data extraction as appropriate based on individual site IRB requirements (Additional file 1: Table S1). Data were analyzed using descriptive statistics and presented as mean ± standard deviation (±SD) or percentages.

## Results

Data were collected for patients with gram-positive osteomyelitis from 39 participating centers. A total of 152 patients with osteomyelitis were enrolled in the CAPTURE study during years 3 and 4 (74 and 78 patients, respectively). Of the 152 patients, 150 (98.7%) were evaluable; one patient had an indeterminate outcome and was not evaluated per protocol, the second patient was not evaluated for reasons unspecified. Most patients were male (102/150; 68.0%) and white (99/150; 66.0%) or black/African American (37/150; 24.7%; Table 1). Mean (±SD) age of evaluable patients was 59.2 (±15.4) years (median 60 y; range 18–92 y) and body mass index (BMI) was 29.7 (±8.3) kg/m^2^.

**Table 1:** Patient Demographics and Baseline Characteristics

Characteristic	CAPTURE (*N* = 150)
Male, n (%)	102 (68.0)
Mean (±SD) age, y	59.2 (±15.4)
Ethnicity, n (%)	
Hispanic/Latino	14 (9.3)
Not Hispanic/Latino	134 (89.3)
Race, n (%)	
White	99 (66.0)
Black or African American	37 (24.7)
Asian	1 (0.7)
American Indian or Alaska native	4 (2.7)
Native Hawaiian or Pacific Islander	0
Mean (±SD) BMI, kg/m^2^	29.7 (±8.3)
Mean (±SD) temperature, °C	37.0 (±0.6)

*BMI* body mass index, *SD* standard deviation

Disease characteristics and the past illnesses of the evaluable osteomyelitis patients from CAPTURE are summarized in Table 2. Osteomyelitis was associated with hardware in 32/150 patients (21.3%). Secondary bacteremia was observed in 25/150 osteomyelitis patients (16.7%). The most common primary sites of infection were as follows: 84/150 (56.0%) foot, 24/150 (16.0%) spine, 18/150 (12.0%) leg or thigh, and 13/150 (8.7%) hip. Mean (±SD) white blood cell count at baseline was 9703.4 (±4228.5) cells/mm^3^. The majority of osteomyelitis patients had been previously diagnosed with DM or PAD (89/150; 59.3%). During ceftaroline fosamil therapy, 22/150 patients (14.7%) underwent surgery, including 10/150 patients (6.7%) who required amputation.

**Table 2:** Disease Characteristics, Past Illnesses, and Surgical History

Category	CAPTURE (*N* = 150)
Mean (±SD) time from diagnosis to discharge, d	10.1 (±42.0)
Disease characteristics, n (%)	
Associated with hardware	32 (21.3)
Associated complications^a^	
Any	132 (88.0)
ABSSSI	99 (66.0)
Secondary bacteremia	25 (16.7)
Other	18 (12.0)
Primary site of infection^a^	
Foot	84 (56.0)
Spine	24 (16.0)
Leg or thigh	18 (12.0)
Hip	13 (8.7)
Other	17 (11.3)
Laboratory results,^b^	
Mean (±SD) total white blood cell count, cells/mm^3^	9703.4 (±4228.5)
Mean (±SD) serum creatinine, mg/dL	1.6 (±2.0)
Mean (±SD) hemoglobin A1c^c^	8.1 (±2.1)
Past illnesses,^a^ n (%)	
DM	87 (58.0)
PAD	23 (15.3)
DM or PAD	89 (59.3)
DM and PAD	21 (14.0)
Current or recent IV drug use	2 (1.3)
Surgical history during CPT-F therapy,^a^ n (%)	
Amputation	10 (6.7)
Incision and drainage	4 (2.7)
Debridement	3 (2.0)
Other	5 (3.3)

*ABSSSI* acute bacterial skin and skin structure infection, *CPT-F* ceftaroline fosamil, *DM* diabetes mellitus, *IV* intravenous, *PAD* peripheral arterial disease, *SD* standard deviation

^a^Categories not mutually exclusive

^b^Before treatment with CPT-F

^c^Measurements taken 2 months before treatment through the end of treatment

Pathogens isolated from osteomyelitis patients are summarized in Table 3. MRSA was the most common, isolated from 93/150 evaluable patients (62.0%). A vancomycin MIC ≤1 was identified in 59/92 MRSA isolates (64.1%) tested for vancomycin susceptibility. Ceftaroline susceptibility was tested in 19 MRSA isolates; a ceftaroline MIC ≤1 was identified in 17/19 MRSA isolates tested (89.4%). The 1 methicillin-sensitive *S aureus* (MSSA) pathogen isolated was tested for susceptibility to ceftaroline and had a ceftaroline MIC < 1 (100%).

**Table 3:** Pathogens Isolated

Pathogen, ^a^ n (%)	CAPTURE (*N* = 150)
MRSA	93 (62.0)
Vancomycin MIC ≤1^b^	59 (64.1)
MSSA	21 (14.0)
Coagulase-negative staphylococci	20 (13.3)
*Streptococcus agalactiae*	18 (12.0)
*Escherichia coli*	4 (2.7)
Other	20 (13.3)

*MIC* minimum inhibitory concentration, *MRSA* methicillin-resistant *Staphylococcus aureus*, *MSSA* methicillin-susceptible *Staphylococcus aureus*

^a^Multiple pathogens were identified in 22/150 patients (14.7%)

^b^Percentage determined using the number of pathogens with MIC testing performed (*n* = 92)

Most patients received ceftaroline fosamil as monotherapy (100/150; 66.7%) at a dose of 600 mg (117/150; 78.0%). Ceftaroline fosamil was typically administered every 12 h (143/150 patients; 95.3%); mean (±SD) duration of ceftaroline fosamil dosing was 8.0 (±7.2) days (median, 6.0 d; range, 2–45 d; Table 4). Of the 50/150 patients (33.3%) who received ceftaroline fosamil as concurrent therapy, 18/150 (36.0%) received metronidazole. Overall, 76% of patients (114/150) received antibiotics before ceftaroline fosamil therapy, of which vancomycin was the most commonly administered (81/150; 54.0%). Of the approximately 71% of patients (106/150) with osteomyelitis who received antibiotics subsequent to ceftaroline fosamil therapy, 35/150 (23.3%), 14/150 (9.3%), and 14/150 (9.3%) received ceftaroline fosamil, daptomycin, and vancomycin, respectively. Antibiotic categories are not mutually exclusive and patients may have been included in more than one category.

**Table 4:** Ceftaroline Fosamil Therapy

Category, n (%)	CAPTURE *N* = 150
Mean (±SD) duration of dosing, d	8.0 (±7.2)
Median (range) duration of dosing, d	6.0 (2–45)
CPT-F dose per treatment^a^	
200 mg	11 (7.3)
300 mg	10 (6.7)
400 mg	23 (15.3)
600 mg	117 (78.0)
CPT-F frequency^a^	
Every 6 h	0
Every 8 h	10 (6.7)
Every 12 h	143 (95.3)
Every 24 h	1 (0.7)
CPT-F used as monotherapy	100 (66.7)
CPT-F used as concurrent therapy	50 (33.3)
Prior antibiotics administered^a^	
Vancomycin	81 (54.0)
Piperacillin with tazobactam	28 (18.7)
Daptomycin	26 (17.3)
Concurrent antibiotics administered^ab^	
Metronidazole	18 (12.0)
Vancomycin	8 (5.3)
Daptomycin	7 (4.7)
Subsequent antibiotics administered^ab^	
CPT-F	35 (23.3)
Daptomycin	14 (9.3)
Vancomycin	14 (9.3)

*CPT-F* ceftaroline fosamil, *SD* standard deviation

^a^Categories not mutually exclusive

^b^Top 3 most commonly received antibiotics for this category

**Fig. 1:**
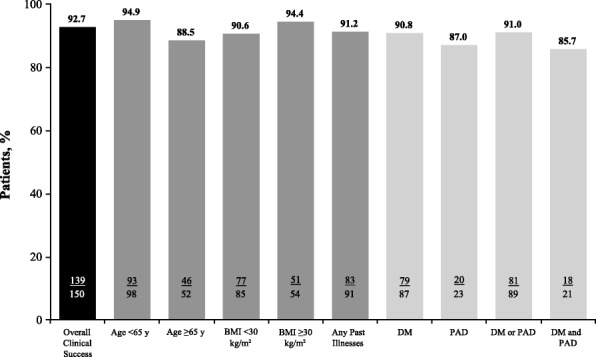
Clinical success after ceftaroline fosamil therapy by relevant demographics and past illnesses*. *Patients may be in more than one category. BMI = body mass index, DM = diabetes mellitus, PAD = peripheral arterial disease

Clinical success of ceftaroline fosamil therapy was observed in 139/150 patients (92.7%; summary provided in Fig. 1). Clinical success rate by BMI were 51/54 (94.4%; BMI ≥30 kg/m^2^) and 77/85 (90.6%; BMI < 30 kg/m^2^). Patients with DM or PAD experienced clinical success approximately equal to the overall osteomyelitis population (81/89; 91.0%). Clinical success rates by past illness (DM or PAD) and infection site are listed in Table 5. Patients with osteomyelitis who had diabetes and a primary infection site in the foot had a clinical success rate of 89.4% (59/66); similarly, patients with osteomyelitis who had peripheral arterial disease and a primary infection site in the foot had a clinical success rate of 89.5% (17/19).

**Table 5:** Clinical Success Rates After Ceftaroline Fosamil Therapy by Past Illness and Infection Site

Primary site of infection,^a^ n/N (%)	DM	Non-DM	PAD	Non-PAD
Foot	59/66 (89.4)	16/18 (88.9)	17/19 (89.5)	58/65 (89.2)
Spine	8/9 (88.9)	15/15 (100)	1/1 (100)	22/23 (95.7)
Leg or thigh	8/8 (100)	10/10 (100)	1/1 (100)	17/17 (100)
Hip	1/1 (100)	12/12 (100)	0	13/13 (100)
Other^b^	4/5 (80.0)	11/12 (91.7)	1/2 (50.0)	14/15 (93.3)

*DM* diabetes mellitus, *PAD* peripheral arterial disease

^a^Categories not mutually exclusive

^b^Other includes head, thorax, arm, forearm, shoulder, hand, and pelvis

**Fig. 2:**
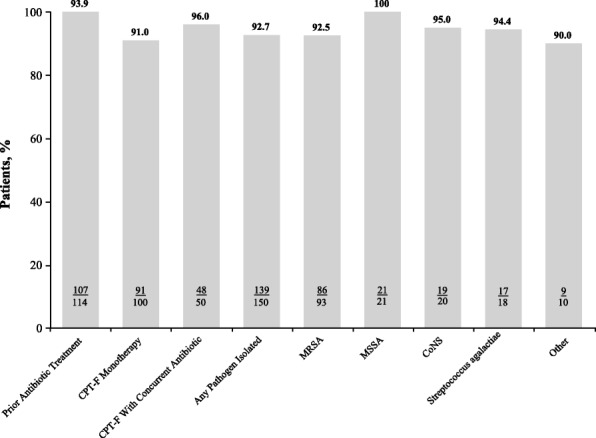
Clinical success rates by antibiotic treatment and pathogen isolated*. *Patients may be in more than one category. CoNS = coagulase-negative staphylococci, CPT-F = ceftaroline fosamil, MRSA = methicillin-resistant *Staphylococcus aureus*, MSSA = methicillin-susceptible *Staphylococcus aureus*

The clinical success rate was high regardless of whether patients had prior antibiotic therapy (107/114; 93.9%]), received ceftaroline fosamil concurrently with other antibiotic therapy (48/50; 96.0%), or as monotherapy (91/100; 91.0%; Fig. 2). Clinical success rates by dosing frequency were 90.0% (9/10), 93.0% (133/143), and 100% (1/1) for patients receiving ceftaroline fosamil every 8, 12, or 24 h, respectively. No patients received ceftaroline fosamil treatment every 6 h. Clinical success was also high in patients who were infected with MRSA, MSSA, or multiple pathogens (86/93 [92.5%], 21/21 [100%], and 21/22 [95.0%], respectively; Fig. 2). Two patients out of 150 (1.3%) discontinued ceftaroline fosamil therapy because of AEs (one case of possible acute interstitial nephritis leading to renal failure and one case of maculopapular rash).

## Discussion

Patients with gram-positive osteomyelitis enrolled in the CAPTURE study exhibited a range of characteristics that are representative of patients commonly treated for osteomyelitis in the clinic, including advanced age, high body mass, past illnesses that include DM and/or PAD, and infection with MRSA. Overall, clinical success rates were high with ceftaroline fosamil therapy. High clinical success rates were also observed in patients with multiple risk factors, including DM and/or PAD. Additionally, high rates of clinical success were observed regardless of age (< 65 or ≥ 65 years), BMI (< 30 or ≥ 30 kg/m^2^), pathogens isolated, or whether ceftaroline fosamil was used as monotherapy, with concurrent antibiotics, or after prior antibiotic treatment.

Data from this study provide information regarding the use of ceftaroline fosamil in clinical practice for treatment of osteomyelitis. Clinical success rates reported here are similar to the reported success rate (94.4%; 67/71) from a retrospective study of patients with osteomyelitis (bone and joint infection) treated with ceftaroline fosamil [18]. A recent analysis of the clinical success rate with ceftaroline fosamil therapy among patients with diabetic foot infection enrolled in CAPTURE reported similar success rates overall (81.1%; 163/201), among patients with DM only (85.8%; 103/120) or DM and PAD (74.1%; 60/81), and among obese patients (BMI ≥30 kg/m^2^; 88.9% [96/108]) [19]. The results reported here for obese versus nonobese patients also support the conclusion of a previous study that found that dosage adjustment for ceftaroline fosamil based on body weight was not necessary in patients with adequate renal function [21]. In two recent case reports, clinical success was observed with 4 to 6 weeks of ceftaroline fosamil therapy for osteomyelitis using dosing schedules (600 mg twice daily) similar to that used for the majority of patients reported here [16, 17].

Discontinuation of ceftaroline fosamil in this study due to an AE was rare. These results were similar to those from an analysis of ceftaroline fosamil in the treatment of complicated skin and skin structure infections [22] and community acquired pneumonia [23], but differ from a retrospective chart review in which ceftaroline fosamil therapy was discontinued in 75% of patients (9/12) because of AEs (predominantly hematologic toxicities; median duration of therapy, 22 days) [24], and two case reports in which two patients who received ceftaroline fosamil developed neutropenia (one with agranulocytosis) after 21 and 32 days, respectively [25, 26]. This disparity in AE frequency may be due, at least in part, to the shorter duration of ceftaroline fosamil therapy received by patients in this analysis.

This study shares the general limitations of retrospective registry studies and did not include a comparator arm. The population size was also somewhat limited. Because this study was designed to capture use of ceftaroline fosamil in the acute care setting and not the complete treatment course for each patient, the duration of treatment was shorter than typically used for treatment of osteomyelitis. Due to the method of data collection, interpretation of the findings is also limited by the ability to pinpoint exactly which antibiotic was given in a specific time frame. In addition, this analysis does not account for the potential disparity between study definition of clinical success and infection cure. Outcomes were also determined by individual physician assessment and definitions were not standardized throughout the registry. As with any antibiotic treatment, resistance to treatment may develop.

Treatment of osteomyelitis may be challenging because of the presence of a biofilm in patients with implanted devices, the relatively high frequency of relapses, and the need to treat for longer periods to allow for the revascularization of infected bone. The addition of ceftaroline fosamil to the clinical options for the treatment of osteomyelitis may hold a number of potential benefits because of the combination of its bone penetration properties (comparable with other agents often used in osteomyelitis therapy) [8, 12], general tolerability [13, 14], and ease of monitoring (not needed for target levels; AEs can be monitored with routine lab tests). In addition, ceftaroline fosamil may provide another option for the treatment of osteomyelitis caused by MRSA.

## Conclusions

These results demonstrate that ceftaroline fosamil is effective for the treatment of gram-positive osteomyelitis in a real-world setting, regardless of risk factors, antibiotic treatment history, or pathogen isolated. Taken together with previously reported bone penetration data and case reports, these data also suggest that ceftaroline fosamil may be considered among clinical options for the treatment of osteomyelitis. Additional studies to further assess efficacy and safety of ceftaroline fosamil in the treatment of osteomyelitis are warranted.

## Additional file

Additional file 1: **Table S1**. Site numbers and Institutional Review Board Names and Addresses (PDF 40 kb)

## Abbreviations

ABSSSI: acute bacterial skin and skin structure infection
AE: adverse event
BMI: body mass index
CANVAS: CeftAroliNe Versus VAncomycin in Skin and Skin Structure Infections
CAPTURE: Clinical Assessment Program and Teflaro® Utilization REgistry
CoNS: coagulase-negative staphylococci
CPT-F: ceftaroline fosamil
DM: diabetes mellitus
IV: intravenous
MIC: minimum inhibitory concentration
MRSA: methicillin-resistant *Staphylococcus aureus*
MSSA: methicillin-susceptible *Staphylococcus aureus*
PAD: peripheral arterial disease
PBP: penicillin-binding protein
SD: standard deviation
