# Supplementary material for: Use of Ceftaroline Fosamil in Osteomyelitis: CAPTURE Study Experience
Source: BMC Infect Dis. 2019 Feb 21;19:183. doi: 10.1186/s12879-019-3791-z (PMC6385445; doi:10.1186/s12879-019-3791-z)
Supplement: Supplementary file 1 — Table S1. Site numbers and Institutional Review Board Names and Addresses (PDF 40 kb) [file 12879_2019_3791_MOESM1_ESM.pdf]

Supplementary Table 1. Site numbers and Institutional Review Board Names and Addresses

Site Number	Institutional Review Board Names
**101**	Medical Center of Central Georgia Institutional Review Board<br>777 Hemlock Street<br>Macon, GA 31201
**102**	Office of Bay Medical Institutional Review Board<br>615 No. Bonita Ave.<br>Panama City, FL 32401
**104**	Promedica Health System Institutional Review Board<br>2142 N. Cove Blvd.<br>Toledo, OH 43606
**105**	Summa Health System, Office of Research Administration<br>525 East Market Street, Suite G-1<br>Akron, OH, 44304
**106**	Human Research Protections Program, McLaren Health Care Corporation<br>1198 N. Belsay Road<br>Building #1,<br>Burton, MI 48509
**107**	Christus Spohn Health System Institutional Review Board<br>919 Hidden Ridge<br>Irving Texas, 75038
**108**	LRG Healthcare Institutional Review Board<br>80 Highland Street<br>Laconia, NH 03246
**109**	Eastern Idaho Regional Medical Center Institutional Review Board<br>3100 Channing Way<br>Idaho Falls, ID 83404
**113**	Covenant Health System Institutional Review Board<br>3615 19th Street<br>Lubbock, TX 79410
**127**	Jamaica Hospital Medical Center Institutional Review Board<br>Van Wyck Expressway<br>Jamaica, NY 11418
**135**	Quorum Review IRB<br>1601 Fifth Avenue<br>Suite 1000<br>Seattle, WA 98101
**148**	South Texas Health System SPI Committee<br>1102 W. Trenton<br>Edinburg, TX 78539
**163**	University of Texas Health Science Center at Houston<br>6410 Fannin Street<br>Suite 1100<br>Houston, Texas 77030
**183**	Quorum Review IRB

	1601 Fifth Avenue<br>Suite 1000<br>Seattle, WA 98101
**208**	Blount Memorial Hospital Investigational Research and Review Committee<br>907 E. Lamar Alexander Pkwy.<br>Maryville, TN 37804-5016
**239**	Sparrow Health System Institutional Review Board<br>Office of Research And Oversight Compliance<br>1215 E. Michigan Avenue<br>PO Box 30480<br>Lansing, MI 48090-7980
**243**	Northern Arizona Healthcare Institutional Review Board<br>1200 North Beaver Street<br>Flagstaff, AZ 68001
**251**	Western Institutional Review Board<br>3535 7th Avenue SW<br>Olympia, WA 98502-5010
**290**	Maimonides Medical Center Institutional Review Board<br>4802 Tenth Avenue<br>Brooklyn, NY 11219
**298**	Quorum Review IRB<br>1601 Fifth Avenue<br>Suite 1000<br>Seattle, WA 98101
**337**	Provena United Samaritans Medical Center Institutional Review Committee<br>812 North Logan Avenue<br>Danville, IL 61832
**343**	Sacred Heart Health System Institutional Review Board<br>5151 N. Ninth Avenue, PO Box 2700,<br>Pensacola, FL 32513
**350**	St. Vincent Research And Regulatory Affairs Department<br>8402 Harcourt Road, Suite 120<br>Indianapolis, IN 46260
**367**	Baptist Health South Florida Institutional Review Board<br>8900 North Kendall Drive<br>Miami, FL 33176-2197
**384**	Tufts Health Sciences Campus Institutional Review Board<br>800 Washington Street, Box 817<br>Boston, MA 02111
**400**	Quorum Review IRB<br>1601 Fifth Avenue<br>Suite 1000<br>Seattle, WA 98101
**412**	Georgetown University Institutional Review Board<br>3900 Reservoir Rd NW, MedDent SW104<br>Washington DC, 20007
**420**	Saint Michaels Medical Center Institutional Review Board<br>111 Central Avenue

	Newark, NJ, 07102
**429**	Partners Human Research Committee<br>116 Huntington Avenue, Suite 1002<br>Boston, MA 02116
**430**	Cape Fear Valley Health System Institutional Review Board<br>1638 Owen Drive<br>Fayetteville, NC 28304
**436**	Mount Sinai Medical Center Institutional Review Board<br>4300 Alton Rd.<br>Miami Beach, FL 33140
**450**	Committee on Clinical Investigation Beth Israel Deaconess Medical Center<br>330 Brookline Avenue<br>Boston, MA 02215
**453**	Quorum Review IRB<br>1601 Fifth Avenue<br>Suite 1000<br>Seattle, WA 98101
**457**	Quorum Review IRB<br>1601 Fifth Avenue<br>Suite 1000<br>Seattle, WA 98101
**458**	Mercy Hospital St. Louis Institutional Review Board<br>621 S. New Ballas Road, Suite 6002-B<br>St. Louis, MO 63141
**459**	Oregon Health & Science University Research Integrity Office<br>3181 SW Sam Jackson Park Road<br>Portland, OR 97239-3098
**460**	University of Kansas Medical Center, Human Research Protection Program<br>MS 1032<br>3901 Rainbow Blvd.<br>Kansas City, KS, 66160
**461**	Wayne State University Institutional Review Board<br>87 East Canfield, Second Floor<br>Detroit, MI 48201
**467**	Christus Spohn Health System Institutional Review Board<br>919 Hidden Ridge<br>Irving Texas, 75038
**468**	Wayne State University Institutional Review Board<br>87 East Canfield, Second Floor<br>Detroit, MI 48201
**471**	Quorum Review IRB<br>1601 Fifth Avenue<br>Suite 1000<br>Seattle, WA 98101
**477**	Albert Einstein College of Medicine, Montefiore Medical Center, West Campus Institutuional Review Board<br>Montefiore Medical Center<br>3308 Rochambeau Avenue<br>Bronx, NY 10467
**479**	Texas Health Resources Institutional Review Board

	612 E. Lamar Blvd., Suite 1212<br>Arlington, TX 76011
**501**	Mercer University Institutional Review Board<br>1550 College Street, MUSM-MHS Suite 2<br>Macon, GA 31207-0001
**525**	Hartford Healthcare Institutional Review Board<br>80 Seymour Street, P.O. Box 5037<br>Hartford, CT 06102-5037
**536**	Quorum Review IRB<br>1601 Fifth Avenue<br>Suite 1000<br>Seattle, WA 98101
**557**	Quorum Review IRB<br>1601 Fifth Avenue<br>Suite 1000<br>Seattle, WA 98101
**559**	Creighton University IRB<br>2500 California Plaza<br>Omaha, NE 68178
**562**	University of Cincinnati Institutional Review Board<br>2600 Clifton Ave.<br>Cincinnati, OH 45221
**563**	St. John Hospital and Medical Center Institutional Review Board<br>19251 Mack Avenue, Suite 340<br>Gross Pointe Woods, MI 48236
**589**	University of Missouri Adult Health Sciences Institutional Review Board<br>5319 Rockhill Road<br>Kansas City, MO 64108
**627**	DeKalb Medical Institutional Review Board<br>2701 North Decatur Rd.<br>Decatur, GA 30033
**662**	Henry Ford Health System Institutional Review Board<br>Research Administration<br>CFP Basement 046<br>2799 West Grand Boulevard<br>Detroit, MI 48202-2689
**665**	Western Institutional Review Board<br>3535 7th Avenue SW<br>Olympia, WA 98502-5010
**667**	University of Tennessee Institutional Review Board<br>960 East Third Street, Suite 102<br>Chattanooga, TN 37403
**668**	Huntsville Hospital Institutional Review Board<br>101 Silvey Road, Huntsville, AL 35801
**676**	University of Maryland, Baltimore Institutional Review Board<br>22 South Greene Street<br>University of Maryland Medical Center Room, S10D13<br>Baltimore, MD 21201
**677**	University of Tennessee Health Science Center Institutional Review Board<br>910 Madison Avenue, Suite 600

	Memphis, TN 38163
**678**	University of Utah Institutional Review Board<br>75 South 2000<br>East Salt Lake City, UT<br>84112
**700**	Columbia Memorial Hospital Institutional Review Board<br>71 Prospect Avenue<br>Hudson, NY 12534
**727**	Stanford University Institutional Review Board<br>Box 5579<br>Stanford, CA 94305
**735**	Quorum Review IRB<br>1601 Fifth Avenue<br>Suite 1000<br>Seattle, WA 98101
**738**	Quorum Review IRB<br>1601 Fifth Avenue<br>Suite 1000<br>Seattle, WA 98101
**740**	St. Luke's Health System Institutional Review Board<br>190 East Bannock Street<br>Boise, ID 83712
**741**	Quorum Review IRB<br>1601 Fifth Avenue<br>Suite 1000<br>Seattle, WA 98101
**745**	Chesapeake Institutional Review Board<br>7063 Columbia Gateway Drive, Suite 110<br>Columbia, MD 21046-3403
**749**	Quorum Review IRB<br>1601 Fifth Avenue<br>Suite 1000<br>Seattle, WA 98101
**753**	Quorum Review IRB<br>1601 Fifth Avenue<br>Suite 1000<br>Seattle, WA 98101
**756**	University of Washington Institutional Review Board<br>4333 Brooklyn Avenue NE, Box 359470<br>Seattle, WA 98195-9470
